# Treatment of non-erosive reflux disease and dynamics of the esophageal microbiome: a prospective multicenter study

**DOI:** 10.1038/s41598-020-72082-8

**Published:** 2020-09-16

**Authors:** Chan Hyuk Park, Seung In Seo, Joon Sung Kim, Sun Hyung Kang, Beom Jin Kim, Yoon Jin Choi, Hyo Joo Byun, Jung-Ho Yoon, Sang Kil Lee

**Affiliations:** 1 Department of Internal Medicine, Hanyang University Guri Hospital, Hanyang University College of Medicine, Guri, Republic of Korea; 2 Department of Internal Medicine, Kangdong Sacred Heart Hospital, Hallym University College of Medicine, Seoul, Republic of Korea; 3 Division of Gastroenterology, Department of Internal Medicine, College of Medicine, Incheon St Mary’s Hospital, The Catholic University of Korea, Seoul, Republic of Korea; 4 Department of Internal Medicine, Chungnam National University School of Medicine, Daejeon, Republic of Korea; 5 Department of Internal Medicine, Chung-Ang University College of Medicine, Seoul, Republic of Korea; 6 Department of Internal Medicine, Korea University Guro Hospital, Seoul, Republic of Korea; 7 Department of Internal Medicine, Yonsei Institute of Gastroenterology, Severance Hospital, Yonsei University College of Medicine, Seoul, Republic of Korea; 8 Department of Internal Medicine, Yonsei Institute of Gastroenterology, Severance Hospital, Yonsei University College of Medicine, 50-1 Yonsei-ro, Seoul, 03722 Republic of Korea; 9 Brain Korea 21 PLUS Project for Medical Science, Yonsei University, Seoul, Republic of Korea

**Keywords:** Microbiology techniques, Oesophageal diseases

## Abstract

Non-erosive reflux disease (NERD) pathogenesis has not been thoroughly evaluated. Here, we assessed the response of patients with NERD to proton pump inhibitor (PPI) therapy; changes in the microbiome and biologic marker expression in the esophageal mucosa were also evaluated. Patients with NERD (n = 55) received esomeprazole (20 mg) for eight weeks. The treatment response was evaluated at baseline, week four, and week eight. Esophageal mucosal markers and oropharyngeal and esophageal microbiomes were analyzed in patients who underwent upper gastrointestinal endoscopy at screening (n = 18). Complete and partial response rates at week eight were 60.0% and 32.7% for heartburn, and 61.8% and 29.1% for regurgitation, respectively. The expressions of several inflammatory cytokines, including IL-6, IL-8, and NF-κB, were decreased at week eight. *Streptococcus*, *Haemophilus*, *Prevotella*, *Veillonella*, *Neisseria*, and *Granulicatella* were prevalent regardless of the time-point (baseline vs. week eight) and organ (oropharynx vs. esophagus). The overall composition of oropharyngeal and esophageal microbiomes showed significant difference (*P* = 0.004), which disappeared after PPI therapy. In conclusion, half-dose PPI therapy for eight weeks could effectively control NERD symptoms. The expression of several inflammatory cytokines was reduced in the esophagus, and oropharyngeal and esophageal microbiomes in patients with NERD showed significant difference. However, the microbial compositions in the oropharynx and esophagus were not affected by PPI therapy in this study. Impact of PPI on the microbiome in patients with NERD should be more investigated in future studies.

## Introduction

Non-erosive reflux disease (NERD) is characterized by the presence of typical gastroesophageal reflux disease (GERD) symptoms, including heartburn and regurgitation, associated with pathological acid reflux but the absence of esophageal erosion^1,2^. NERD presents in approximately 70% of patients with GERD^3,4^. Acid-suppressive therapy with proton pump inhibitors (PPIs) is a mainstay in the treatment of NERD^5,6^. However, the therapeutic gain of PPIs in the treatment of NERD is relatively low, compared to the treatment of erosive esophagitis^7^.

Recently, the assumption that NERD and erosive esophagitis represent one continuous disorder within the spectrum of GERD has been challenged because several studies demonstrated differences in the epidemiological features, pathophysiological characteristics, as well as treatment responses of these diseases^3,8–10^. NERD has several differential pathogenic characteristics, including relatively low mucosal permeability, high visceral sensitivity, and common psychological co-morbidity, compared to erosive esophagitis^11^. Nevertheless, our understanding of the pathogenesis of NERD is still incomplete.

Recently, there have been attempts to identify the impact of the microbiome on the development of GERD^12^. The influence of the microbiome on the development of digestive diseases is an emerging issue in gastroenterological research. We know that *Helicobacter pylori* infection is the most important cause of gastric cancer and peptic ulcer diseases^13^. Additionally, *Fusobacterium nucleatum*, which is a periodontal bacteria that is also found in the intestine, has been suggested as a cause of colorectal cancer^14–16^. Although this causal relationship has not been proven, recent studies demonstrated that several bacterial taxa, including *Prevotella*, *Haemophilus*, *Neisseria*, and *Veillonella*, are abundant in the esophagus of patients with GERD^17–19^. Additionally, it has been shown that PPIs may be associated with the composition of the esophageal microbiome^19^.

Until now, however, the esophageal microbiome in patients with NERD has not been investigated. To increase our understanding of NERD, we aimed to evaluate the treatment response to PPIs in patients with NERD and changes in their biologic marker expression and microbial composition before and after PPI therapy. We also compared the esophageal microbiome of the NERD patients with the oropharyngeal microbiome due to the tight regulatory interplay of these microbiomes with each other.

## Results

### Study participants and baseline characteristics

Sixty-six patients were assessed for eligibility for this study between October 2018 and August 2019. After excluding four patients with erosive esophagitis, 62 patients were enrolled in this study. Among them, seven patients withdrew their consent to participate in the study during the study period. As a result, the study finally included 55 patients, for whom treatment response was assessed. Of these 55 patients, 18 who had not been diagnosed with NERD, underwent upper gastrointestinal endoscopy for screening, and oral swab and esophageal mucosal biopsy were performed for these 18 patients.

Baseline characteristics of participants are demonstrated in Table 1. The mean age and proportion of males were 56.1 years and 41.8%, respectively. The proportion of patients with obesity was 38.2%. The frequency and duration were 4.8 /week and 22.5 months for heartburn and 3.6 /week and 23.6 months for regurgitation, respectively. Baseline characteristics of participants who were assessed for biologic markers and microbiomes are also shown in Table 1.

**Table 1:** Baseline characteristics of included patients.

Variable	All patients	Patients with biologic marker and microbial data
N	55	18
Age, year, mean ± SD	56.1 ± 15.3	56.8 ± 15.1
**Sex, n (%)**		
Male	23 (41.8)	10 (55.6)
Female	32 (58.2)	8 (44.4)
BMI, mean ± SD	24.6 ± 3.8	22.8 ± 2.7
Obesity (BMI ≥ 25), n (%)	21 (38.2)	3 (16.7)
**Smoking habit, n (%)**		
Never smoker	40 (72.7)	12 (66.7)
Former smoker	9 (16.4)	3 (16.7)
Current smoker	6 (10.9)	3 (16.7)
**Alcohol consumption, n (%)**		
Never consumption	29 (52.7)	6 (33.3)
Former consumption	3 (5.5)	3 (16.7)
Current consumption	23 (41.8)	9 (50.0)
**Comorbidity, n (%)**		
Hypertension	10 (18.2)	2 (11.1)
Diabetes mellitus	5 (9.1)	0 (0.0)
Old cerebrovascular accident	2 (3.6)	0 (0.0)
Arrythmia	1 (1.8)	0 (0.0)
Other cardiac disease	3 (5.5)	0 (0.0)
Dyslipidemia	11 (20.0)	4 (22.2)
Fatty liver	2 (3.6)	1 (5.6)
History of malignant disease	2 (3.6)	0 (0.0)
**Heartburn**		
Frequency, /week, mean ± SD	4.8 ± 4.5	18.4 ± 27.4
Duration, month, mean ± SD	22.5 ± 58.2	5.7 ± 4.6
**Regurgitation**		
Frequency, /week, mean ± SD	3.6 ± 3.4	22.1 ± 36.5
Duration, month, mean ± SD	23.6 ± 59.8	5.2 ± 4.4

BMI, body mass index; SD, standard deviation.

### Treatment response

**Figure 1:**
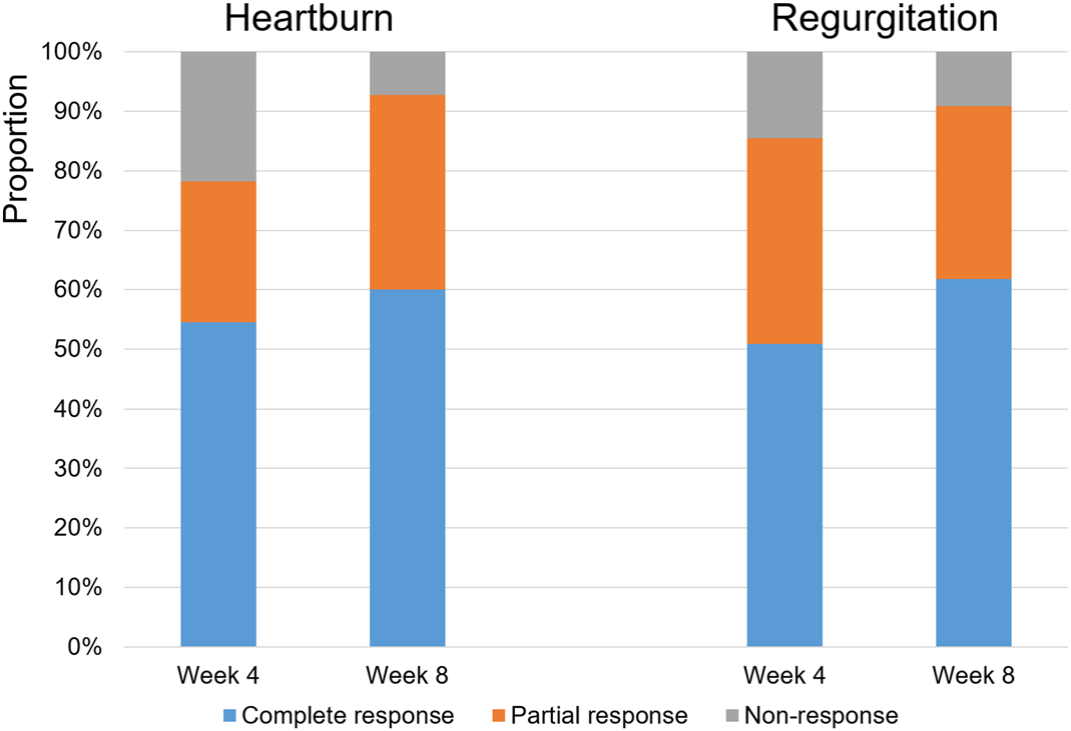
Proton pump inhibitor treatment response for typical gastroesophageal reflux diseases. At week four, the complete and partial response rates for heartburn were 54.5% and 23.6%, respectively, and the complete and partial response rates for regurgitation were 50.9% and 34.5%, respectively. After PPI treatment for eight weeks, the complete response tended to increase for both heartburn and regurgitation compared to those at week four. PPI, proton pump inhibitor.

At week four, the complete and partial response rates were 54.5% and 23.6%, respectively, for heartburn and 50.9% and 34.5%, respectively, for regurgitation (Fig. 1). The complete response tended to increase for both heartburn and regurgitation at week eight compared to that at week four (heartburn at week eight: complete, 60.0%; partial, 32.7%; regurgitation at week eight: complete, 61.8%; partial, 29.1%).

Table 2 shows the data for patient adherence to PPIs and changes in symptom scores obtained using the patient assessment of upper gastrointestinal symptom severity index (PAGI-SYM) and the hospital anxiety and depression scale (HAD) questionnaires. The PAGI-SYM score, based on a six-point Likert scale (from zero to five), was 1.85 ± 1.43 for heartburn and 2.18 ± 1.45 for regurgitation at baseline, both of which decreased at week four (*P* < 0.001 for both). The adherence rate was 97.5 ± 5.2% for the first four weeks, and 96.8% ± 5.8% for the next four weeks (between week four and week eight). The PAGI-SYM score for heartburn at week eight was lower than that at baseline; however, there was no significant difference between the scores at week four and week eight (week four, 0.65 ± 0.93; week eight, 0.54 ± 0.80; *P* = 0.381). The PAGI-SYM score for regurgitation at week eight further decreased compared to that at week four (week four, 0.73 ± 0.93; week eight, 0.50 ± 0.38; *P* = 0.035).

**Table 2:** Changes in symptom scores after proton pump inhibitor administration.

Variable	Baseline	Week 4	*P*-value (vs. baseline)	Week 8	*P*-value (vs. baseline)	*P*-value (vs. week 4)
**PAGI-SYM (0–5)**						
Heartburn/regurgitation						
Heartburn	1.85 ± 1.43	0.65 ± 0.93	< 0.001	0.54 ± 0.80	< 0.001	0.381
Regurgitation	2.18 ± 1.45	0.73 ± 0.93	< 0.001	0.50 ± 0.38	< 0.001	0.035
Nausea/vomiting	0.97 ± 1.09	0.32 ± 0.52	< 0.001	0.23 ± 0.66	< 0.001	0.396
Early satiety	1.49 ± 1.03	0.84 ± 0.72	< 0.001	0.59 ± 0.64	< 0.001	0.001
Bloating	1.35 ± 1.28	0.74 ± 0.91	< 0.001	0.48 ± 0.85	< 0.001	0.018
Upper abdominal pain	1.72 ± 1.46	0.76 ± 1.13	< 0.001	0.56 ± 0.85	< 0.001	0.166
Lower abdominal pain	0.85 ± 1.21	0.60 ± 1.00	0.069	0.45 ± 0.74	0.004	0.107
HAD (0–3)						
Anxiety	0.76 ± 0.49	0.57 ± 0.42	< 0.001	0.50 ± 0.38	< 0.001	0.038
Depression	0.81 ± 0.44	0.67 ± 0.46	0.003	0.58 ± 0.44	< 0.001	0.019
Adherence rate (%)		97.5 ± 5.2		96.8 ± 5.8		0.435

PAGI-SYM, HAD, and adherence rate were presented as mean with standard deviation.

Adherence rate was defined as the number of drugs taken divided by the number of drugs to be taken.

PAGI-SYM, patient assessment of upper gastrointestinal symptom severity index; HAD, hospital anxiety and depression scale.

PAGI-SYM scores for nausea/vomiting, early satiety, bloating, upper abdominal pain, and lower abdominal pain decreased at week four compared to those at baseline, and the scores for early satiety and bloating further decreased at week eight compared to those at week four. Additionally, HAD scores for both anxiety and depression decreased from baseline to week four, as well as from week four to week eight.

### Biologic markers in esophageal mucosa

Real-time quantitative reverse transcription polymerase chain reaction (qRT-PCR) was performed to assess the expression of mRNAs encoding biologic markers in 18 patients who received upper gastrointestinal endoscopy during screening. The complete and partial response rates for eight weeks of PPI therapy among these 18 patients were 55.6% and 38.9%, respectively, for heartburn, and 50.0% and 33.3%, respectively, for regurgitation (Table S1).

**Figure 2:**
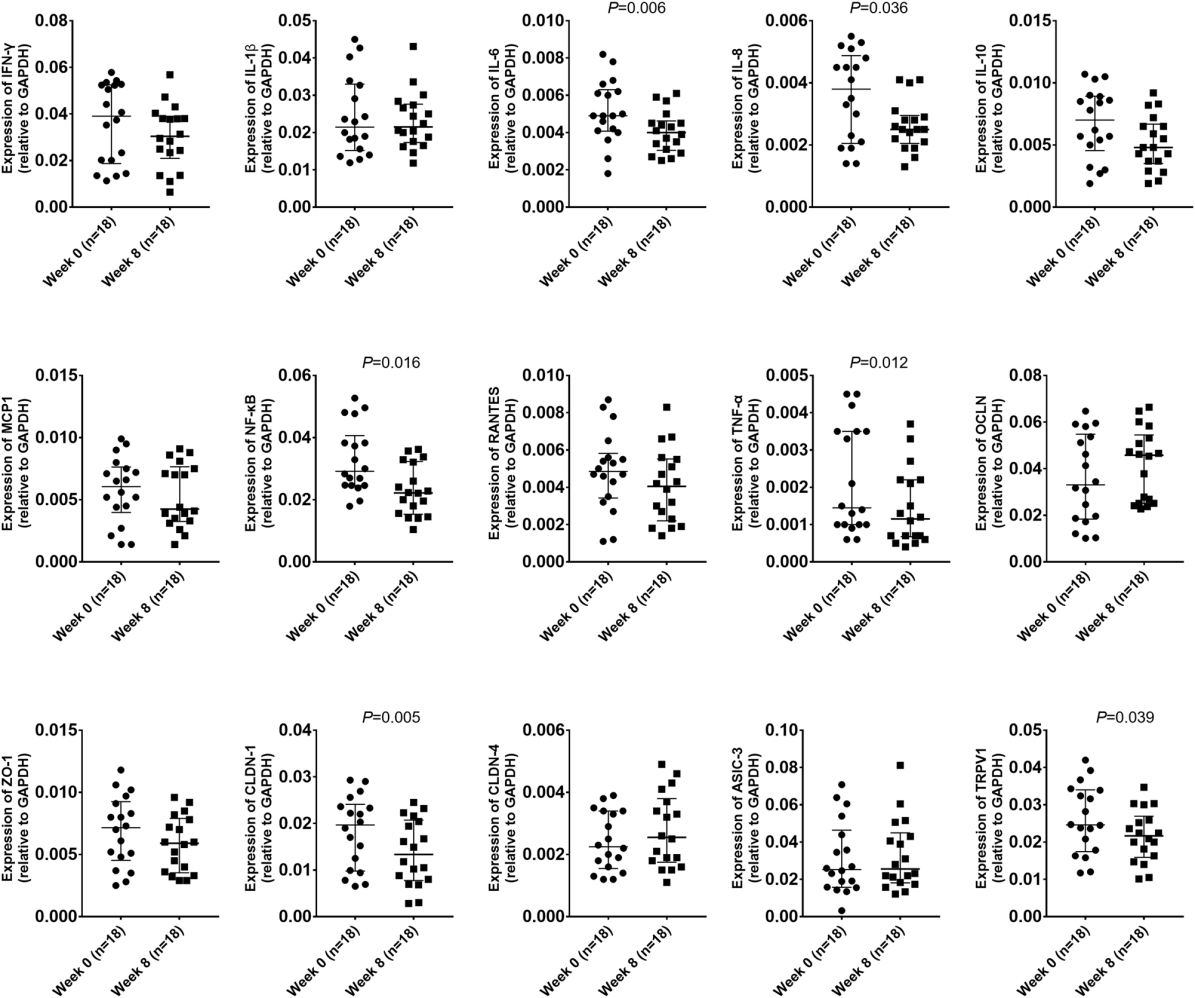
Changes in the expression of biologic markers in the esophageal mucosa after eight weeks of proton pump inhibitor administration. Nine inflammatory markers (IFN-γ, IL-1β, IL-6, IL-8, IL-10, MCP1, NF-κB, RANTES, and TNF-α), four permeability markers (OCLN, ZO-1, CLDN-1, and CLDN-4), and two hypersensitivity markers (ASIC-3 and TRPV1) were assessed. The expression levels of IL-6, IL-8, NF-κB, TNF-α, CLDN-1, and TRPV1 showed statistically significant decrease after PPI administration. All nine inflammatory markers, except IL-1β, showed a tendency for decreased expression after PPI administration. Lines indicate the medians with interquartile ranges. *P*-values were indicated only if the comparisons were significant. PPI, proton pump inhibitor.

Figure 2 shows changes in the expression of biologic markers in the esophageal mucosa after PPI therapy for eight weeks. Of the 15 tested markers, the expression of mRNAs encoding IL-6, IL-8, NF-κB, TNF-α, CLDN-1, and TRPV1 showed statistically significant decreases after PPI administration. All nine inflammatory makers except IL-1β showed a tendency for decreased expression after PPI administration. In the subgroup analysis between patients with complete response for both heartburn and regurgitation (n = 8) and those with partial response or non-response (n = 10), no difference in the expression of biologic markers was observed except for MCP1 [median (interquartile range): complete response 0.008 (0.005–0.009), partial or non-response 0.004 (0.002–0.005); *P* = 0.016].

### Oropharyngeal and esophageal microbiomes

Oropharyngeal and esophageal microbiome analyses were performed in the 18 patients who underwent upper gastrointestinal endoscopy; however, several patients were excluded due to insufficient amounts of extracted DNA for microbiome analyses (n = 1 excluded for week eight oropharyngeal microbiome analysis, n = 6 excluded for baseline esophageal microbiome analysis, and n = 8 excluded for week eight esophageal microbiome analysis).

The number of operational taxonomic units (OTUs) and the Chao1 index tended to be lower in the esophagus than in the oropharynx at baseline as well as at week eight; however, there was no significant difference, except in the Chao1 index, between oropharynx and esophagus at week eight (Figure S1). Shannon and inverse Simpson indices were similar regardless of organ (oropharynx vs. esophagus) and time-point (baseline vs. week eight).

**Figure 3:**
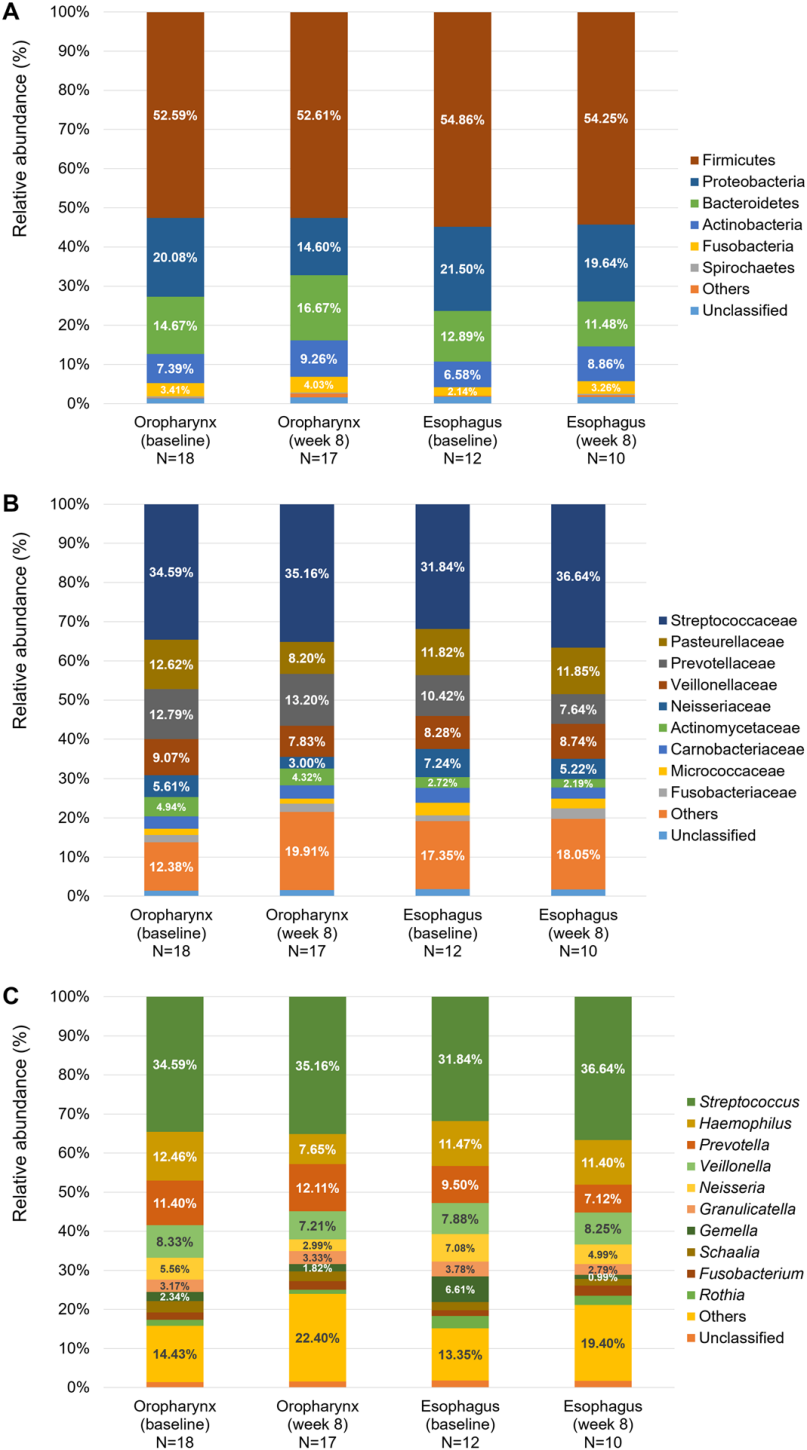
Microbial compositions according to the organ (oropharynx vs. esophagus) and time-point (baseline vs. week eight) at the (**A**) phylum level, (**B**) family level, and (**C**) genus level. At the phylum level, the common bacterial taxa were Firmicutes, Proteobacteria, and Bacteroidetes regardless of organ and time-point. At the genus level, *Streptococcus*, *Haemophilus*, *Prevotella*, *Veillonella*, *Neisseria*, and *Granulicatella* were commonly identified. Values in the bars represent the relative abundance of individual taxa.

**Figure 4:**
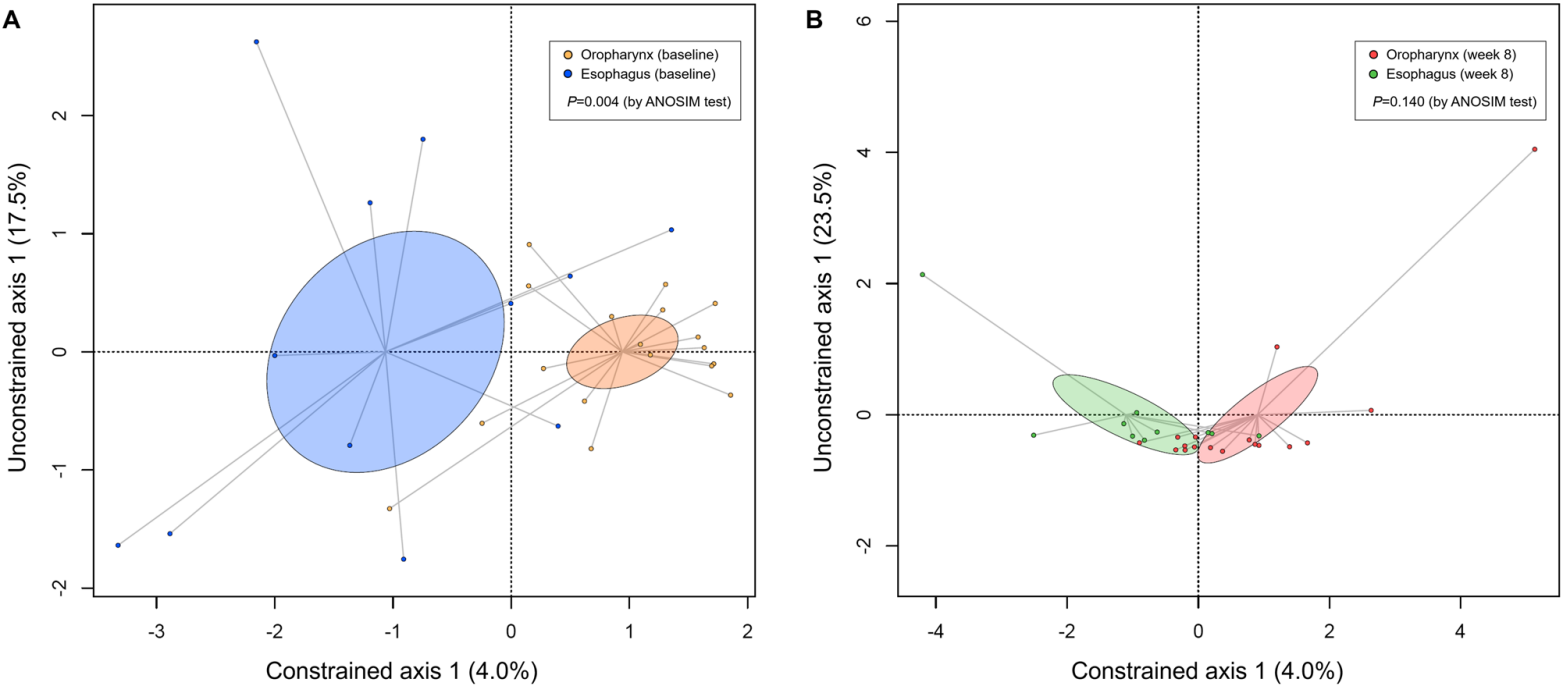
Principal component analysis of the microbiome according to the organ (oropharynx vs. esophagus) at (**A**) baseline and (**B**) week eight. At baseline, the oropharynx had different microbial composition compared to the esophagus (*P* = 0.004 by the ANOSIM test). On the contrary, at week eight, the difference in microbial compositions between the organs (oropharynx vs. esophagus) were not identified (*P* = 0.140 by the ANOSIM test). ANOSIM, analysis of similarities.

Figure 3 demonstrates the compositions of the microbiome by organ and time-point. At the phylum level, the common bacterial taxa were Firmicutes, Proteobacteria, and Bacteroidetes regardless of organ and time-point. At the genus level, *Streptococcus*, *Haemophilus*, *Prevotella*, *Veillonella*, *Neisseria*, and *Granulicatella* were commonly identified. As shown in Fig. 4, at baseline, the overall composition of the oropharyngeal microbiome was different from that of the esophageal microbiome (*P* = 0.004). On the contrary, at week eight, there was no significant difference between the oropharyngeal microbiome and the esophageal microbiome (*P* = 0.140). Additionally, no significant difference was identified between the baseline and week eight in the oropharyngeal microbiome as well as in the esophageal microbiome (*P* = 0.334 and *P* = 0.920, respectively; Figure S2).

As shown in Figure S3, the linear discriminant analysis demonstrated the relatively abundant bacterial taxa in each group. At baseline, Enterobacteriaceae and Chitinophagaceae were more abundant whereas unclassified Clostridiales Family XIII and Methylobacteriaceae were less abundant in the esophageal microbiome than in the oropharyngeal microbiome. At week eight, there was no difference in the relative abundance of bacterial taxa in the esophageal microbiome and those in the oropharyngeal microbiome.

### Correlations between the expression of biologic markers and the relative abundance of bacterial taxa

**Figure 5:**
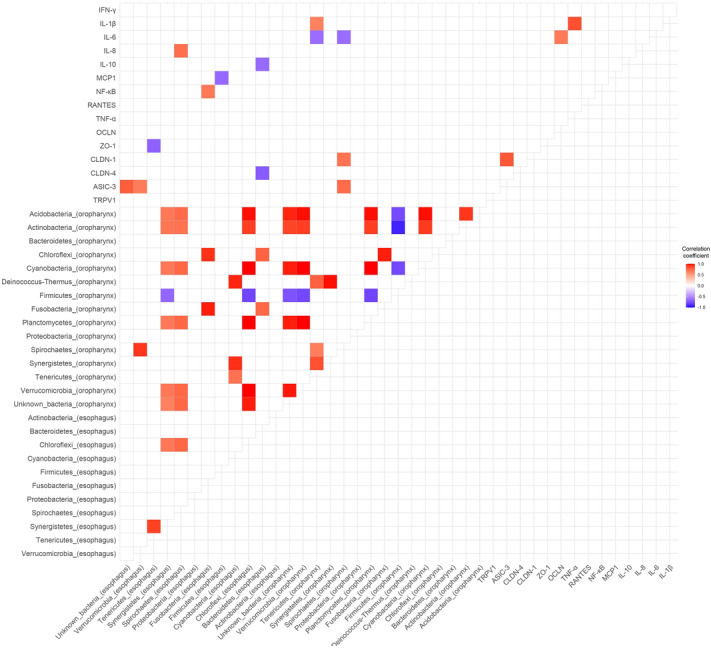
Correlations between the expression of biologic marker and the relative abundance of bacterial taxa (n = 10). The heatmap indicates the correlation coefficients between the expression of biologic markers and the relative abundance of bacterial taxa at the phylum level. Only statistically significant correlations are presented in this heatmap. The correlation was more common between bacterial taxa and other bacterial taxa. NF-κB, IL-1β, and IL-8 were correlated with esophageal Fusobacteria (γ = 0.68, *P* = 0.032), oropharyngeal Tenericutes (γ = 0.64, *P* = 0.048), and esophageal Spirochaetes (γ = 0.72, *P* = 0.018), respectively. γ, correlation coefficient.

Figure 5 shows the correlation coefficients between the expression of biologic markers and the relative abundance of bacterial taxa at the phylum level. Overall, the correlation was commonly observed between some bacterial taxa and other bacterial taxa. However, several biologic markers, including NF-κB, IL-1β, and IL-8, were also correlated with bacterial taxa [NF-κB and esophageal Fusobacteria: correlation coefficient (γ) = 0.68, *P* = 0.032; IL-1β and oropharyngeal Tenericutes: γ = 0.64, *P* = 0.048; IL-8 and esophageal Spirochaetes: γ = 0.72, *P* = 0.018].

## Discussion

In the current study, we evaluated the treatment response to 20 mg of esomeprazole in patients with NERD. As previously shown, NERD is less likely to respond to PPI therapy^7,20,21^. Our study also revealed the non-response rate of PPI administration for four weeks to be 21.8% for heartburn and 14.5% for regurgitation. These results were relatively higher compared to the results of the large-scale study on GERD (including erosive esophagitis) conducted by Eggleston et al.^22^ In that study, the non-response rate was 9.8% for heartburn and 12.4% for regurgitation after four weeks of PPI treatment (20 mg of esomeprazole, which is the same dose as that used in our study). The complete response rate in our study was 54.5% for heartburn and 50.9% for regurgitation, whereas in the study by Eggleston et al. it was 60.6% for heartburn and 60.1% for regurgitation. The relatively lower response rate in our study than that reported by Eggleston et al. may be due to the inclusion of only patients with NERD.

However, the treatment response rate may be increased by prolonged PPI therapy. In our study, the complete response rate at week eight was 60.0% for heartburn and 61.8% for regurgitation, and the non-response rate at week eight was 7.3% for heartburn and 9.1% for regurgitation. These eight-week response rates in this study were similar to the four-week response rates in the study by Eggleston et al., which included both erosive esophagitis and NERD patients. Even in NERD patients, typical symptoms can be effectively controlled by prolonged PPI therapy.

One of the main causes of treatment failure in patients who were presumably diagnosed with NERD is an incorrect diagnosis. Some patients diagnosed with NERD may actually have esophageal hypersensitivity or functional heartburn, rather than true NERD^20^. In the current study, we included patients who showed typical GERD symptoms with high frequency and a relatively long duration. The participants manifested symptoms other than heartburn/regurgitation, such as nausea/vomiting or bloating, with less severity. Although we did not perform a 24 h-ambulatory pH monitoring, we believe that patients with true NERD, rather than esophageal hypersensitivity or functional heartburn, were mainly included in our study. It should be kept in mind that prolonged PPI therapy does not always guarantee successful symptom control in patients who are presumably diagnosed with NERD.

In our study, we also aimed to evaluate changes in biologic markers, which are already known to be related to reflux esophagitis of the esophageal mucosa. Among inflammatory, permeability, and hypersensitivity markers, inflammatory cytokines were mostly reduced by PPI therapy. All inflammatory biomarkers, except IL-1β, decreased after PPI administration, with moderate amplitudes. In our previous study, IL-1β expression did not show any significant difference when ilaprazole, a new class of gastric acid secretion inhibitors, was administered to NERD patients^23^. Among the decreased inflammatory cytokines, the most notable one was NF-κB, which is a pleiotropic regulator of the inducible expression of several genes^24–26^. Constitutively activated NF-κB is commonly identified in various tumors and cancer cell lines^27–30^. Several studies reported that it regulates cell proliferation, tumor development, and cell transformation^25,31^. The expression of NF-κB gradually increases as disease progressed from Barrett’s esophagus to esophageal adenocarcinoma^24,32,33^. In our study, the esophageal expression of NF-κB in patients with NERD was significantly reduced after eight weeks of treatment. Acid suppression by PPI therapy not only improved typical GERD symptoms but also reduced the expression of inflammatory cytokines. Additionally, the expression of NF-κB was significantly correlated with the abundance of esophageal Fusobacteria. Although causality cannot be concluded between Fusobacteria and esophageal inflammation, this may be an interesting finding because Fusobacteria has been known to be related to gingivitis, periodontitis, esophagitis, and esophageal cancer^34,35^.

In addition to NF-κB, we showed that the expression levels of IL-6 and IL-8 reduced after PPI therapy. It is known that NF-κB upregulates various cytokines, including IL-6, leading to an increase in STAT3 signalling^33^. Another study demonstrated that both IL-6 and activated STAT3 were increased in transformed Barrett’s cells^33,36^. IL-8 is a potent activator of NF-κB and is commonly found in esophageal adenocarcinoma^24,37^. Recently, Dunbar et al*.* reported that reflux esophagitis may be caused by pro-inflammatory cytokines rather than chemical injury by gastric acid, bile, and pepsin^38^. PPI therapy has a direct anti-inflammatory effect, independent of its effect on acid secretion^38^. Although the mechanisms of PPI activity in NERD are not exactly known, we found that PPI reduced the inflammation even in NERD patients.

One of the major findings in our study is oropharyngeal and esophageal microbiomes before and after PPI therapy. We found that *Streptococcus* is the most prevalent bacterial taxon in the esophageal microbiome of patients with NERD. It is well known that *Streptococcus* is abundant in the normal as well as diseased esophagus (*i.e.*, GERD or Barrett’s esophagus)^35^. We additionally identified *Haemophilus*, *Prevotella*, *Veillonella*, *Neisseria*, and *Granulicatella* to be common in the esophagus of patients with NERD. These bacterial taxa belong to the type II microbiome, which is suggested as being characteristic of patients with GERD and Barrett’s esophagus by Yang et al.^17^.

The relative abundances of major bacterial taxa were roughly similar between the oropharynx and esophagus of NERD patients, as shown in Fig. 3. Traditionally, microbial flora in the esophagus has been considered to be transient and to have translocated from the oropharynx^39^. In a culture-based study, *Streptococcus* was the most commonly isolated bacteria in the esophagus, as well as the oropharynx^39^. In our study, we found that the relative abundances of major bacterial taxa were similar between the oropharynx and esophagus using 16S rRNA gene sequencing analysis. Most bacterial taxa in the esophagus may be derived from the bacterial populations in the oropharynx even in patients with NERD. Although the abundances of major bacterial taxa seem to be similar between the oropharynx and esophagus, the PCA plot for the baseline microbiome demonstrated that the composition of esophageal microbiome was significantly distinguished from that of oropharyngeal microbiome as shown in Fig. 4A. The relative abundances of minor bacterial taxa at the genus level (designated as “others” in Fig. 3C) was 14.4% for the oropharynx and 22.4% for the esophagus. After PPI therapy, however, there was no significant difference in microbial composition between the oropharynx and esophagus. These findings implied that the relative abundances of minor bacterial taxa in the esophagus of patients with NERD may depend on acid reflux and can be resolved by PPI therapy. These minor bacterial taxa included Enterobacteriaceae, Chitinophagaceae, unclassified Clostridiales Family XIII, and Methylobacteriaceae as was shown by the linear discriminant analysis. However, these findings should be cautiously interpreted because the microbial compositions did not significantly differ between the baseline and week eight in each organ (oropharynx or esophagus).

This is the first study to evaluate the treatment response and changes in the expression of biologic markers and microbial compositions after PPI therapy in patients with NERD. Nevertheless, the current study has several limitations. First, the sample size of the study was relatively small, and only 18 patients were included in the analyses of biologic markers and microbiomes, which were secondary endpoints of our study. Therefore, results of biologic markers and microbial compositions should be cautiously interpreted. Second, the diagnosis of NERD was not confirmed by 24 h-ambulatory pH monitoring. Therefore, patients with esophageal hypersensitivity or functional heartburn rather than true NERD might be included, which is a common limitation in studies similar to this study. However, NERD is commonly diagnosed and treated without pH monitoring in the clinical practice. To reflect the real-world situation, we included patients diagnosed with NERD without pH monitoring. Third, the amount of extracted DNA was insufficient to analyze the microbiome in several esophageal samples although we obtained two biopsy samples for microbiome analysis from all candidates. This may be due to lower abundance of the esophageal microbiome in comparison to that in other environments, such as the large intestine. In our experience, enough DNA can be extracted from four stomach biopsy samples^40^. In subsequent studies on the esophageal microbiome, we will consider obtaining more than two pieces of biopsy samples for adequate analysis. Fourth, our study population did not include healthy individuals. As a matter of fact, we only compared the GERD symptom scores, expression levels of biologic markers, and microbial compositions between two different time points (or between organs in the microbiome analysis). Therefore, it is difficult to determine whether the treatment response and changes in biologic marker expression and microbial composition are entirely due to PPI therapy. The patients with erosive reflux disease were not included in this study. Further studies on different disease status (NERD vs. healthy or erosive reflux disease) may be required. Fifth, dietary intake was not controlled in our study, which might impact the microbial compositions in the oropharynx and esophagus. If the dietary intake had been controlled, a more definitive conclusion may have been reached.

Despite these limitations, our study provides a better understanding of the efficacy of PPI therapy as well as data on biologic marker expression after administration of PPIs, together with analysis of oropharyngeal and esophageal microbiomes in patients with NERD. Half-dose PPI therapy for eight weeks was effective for typical GERD symptom control. PPI therapy also reduced the expression of inflammatory cytokines, including IL-6, IL-8, and NF-κB, in the esophagus. Although major bacterial taxa in the esophagus, including *Streptococcus*, *Haemophilus*, *Prevotella*, *Veillonella*, *Neisseria*, and *Granulicatella*, were similar to those in the oropharynx, the overall microbial composition was different between oropharynx and esophagus before PPI therapy in patients with NERD. However, impact of PPI on the oropharyngeal or esophageal microbiome was not identified in this study. The microbial compositions in the oropharynx and esophagus were not affected by PPI therapy. Impact of PPI on the microbiome in patients with NERD should be more investigated in future studies.

## Methods

### Study design

Our trial was a prospective, multicenter, single-arm study with an eight-week duration (ClinicalTrials.gov NO. NCT03436914). We included patients aged 19 years or older who had been diagnosed with NERD based on the presence of typical symptoms, including heartburn, regurgitation, and the absence of esophageal erosion in the study. Patients who experienced heartburn or regurgitation at least ≥ one day/week for more than three months before screening were included. The exclusion criteria were as follows: (1) active peptic ulcer diseases, (2) previous upper gastrointestinal surgery, and (3) pregnancy or breast-feeding. If patients were receiving antibiotics, acid-suppressive agents, probiotics, prokinetics, mucoprotective agents, aspirin, nonsteroidal anti-inflammatory drugs, or corticosteroids, their medications had to be stopped for at least four weeks before starting the treatment. Written informed consent was obtained from all participants before screening. The Institutional Review Board of the seven participating hospitals in Korea (Severance Hospital, Hanyang University Guri Hospital, Kangdong Sacred Heart Hospital, Incheon St Mary’s Hospital, Chungnam National University Hospital, Chung-Ang University Hospital, Korea University Guro Hospital) approved this study. All methods were carried out in accordance with relevant guidelines and regulations.

Patients who underwent upper gastrointestinal endoscopy within six months of enrolment and who were diagnosed with NERD were exempted from the screening endoscopy. They received 20 mg of esomeprazole (Esomezol, Hanmi Pharm. Co., Ltd., Seoul, Korea) for eight weeks. To evaluate the treatment response of PPIs in NERD, the PAGI-SYM and the HAD questionnaires were completed at baseline (week zero), week four, and week eight^41,42^.

If patients had not been diagnosed with NERD and/or had not undergone upper gastrointestinal endoscopy within six months of enrolment, they underwent screening with upper gastrointestinal endoscopy to rule out organic diseases and erosive esophagitis. Each participant received an oropharyngeal swab to assess the oropharyngeal microbiome, followed by upper gastrointestinal endoscopy. If esophageal erosion was not found upon endoscopy and the participant met other inclusion criteria, four pieces of esophageal mucosa were obtained by a biopsy at 3 cm above the gastroesophageal junction to evaluate biologic markers and the microbiome (two for biologic marker analysis and two for microbiome analysis). Patients also received 20 mg of esomeprazole (Esomezol, Hanmi Pharm. Co., Ltd., Seoul, Korea) for eight weeks and questionnaires (PAGI-SYM and HAD) at baseline (week zero), week four, and week eight. At the end of the eight weeks, the participants received oropharyngeal swabs and upper endoscopy to obtain four additional pieces of esophageal mucosa.

### Study endpoint

The primary endpoint was the treatment response in terms of typical GERD symptoms—heartburn and regurgitation. The secondary endpoints were as follows: (1) the treatment response in terms of other symptoms, including nausea/vomiting, early satiety, bloating, upper and lower abdominal pain, anxiety, and depression; (2) the expression of biologic markers, and (3) the compositions of oropharyngeal and esophageal microbiomes.

### Definition

Treatment responses of typical GERD symptoms were classified into three categories—complete response, partial response, and non-response, based on the PAGI-SYM scores. If the PAGI-SYM score reached zero points, the treatment response was regarded as a complete response. If PAGI-SYM score after PPI administration was lower compared to that at baseline without reaching zero, the treatment response was regarded as a partial response. Non-response was defined as a lack of improvement of the PAGI-SYM score after PPI administration. The patient’s adherence rate to the study medication was defined as the number of drugs taken divided by the number of drugs to be taken. Body mass index (BMI) was calculated as the measured weight (kg) divided by the square of the height (/m^2^), and obesity was defined as BMI ≥ 25 kg/m^2^, which is the proposed cut-off value for the diagnosis of obesity in Asians^43^.

### Evaluation of biologic markers

To evaluate biologic markers in the esophageal mucosa, the biopsy samples were immediately stored in RNA later (Invitrogen, Carlsbad, CA, USA) at the bedside. Fresh tissues in RNA later were maintained at − 80 °C until the measurement of messenger RNA (mRNA) levels. All the samples obtained at different institutes were collected at Severance hospital. RNA was extracted from the esophageal mucosal samples as described in our previous study^23,44^.

For complementary DNA synthesis, total RNA was reverse transcribed using SuperScript II (Invitrogen) following the manufacturer's protocol. qRT-PCR was performed using iQ SYBR Green Supermix (Applied Biosystems Inc., Carlsbad, CA, USA) and a Roche Light Cycler480 Real-Time PCR System (Idaho Technology Inc., Salt Lake City, UT, USA).

The fifteen biologic markers evaluated in this study include inflammatory (IFN-γ, IL-1β, IL-6, IL-8, IL-10, MCP1, NF-κB, RANTES, and TNF-α), permeability (OCLN, ZO-1, CLDN-1, and CLDN-4), and hypersensitivity (ASIC-3 and TRPV1) markers. The expression of biologic markers was calculated using the 2^*-ΔΔCT*^ method, with the expression level of glyceraldehyde-3-phosphate dehydrogenase used for normalization. The primer sequences for qPCR are listed in Table S2.

### Evaluation of the oropharyngeal and esophageal microbiome

DNA extraction from the oropharyngeal and esophageal mucosal samples was performed as described previously^40^. Next, we performed 16S rRNA gene sequencing for identifying microbiome composition. DNA quantification and quality assessment were performed using PicoGreen and Nanodrop, respectively. Input gDNA was amplified using 16S V3–V4 primers. To add multiplexing indices and Illumina sequencing adapters, limited‐cycle amplification was performed. We normalized and pooled the final products using PicoGreen. The library size was verified using TapeStation DNA screentape D1000 (Agilent). We subsequently sequenced DNA using the MiSeq platform (Illumina, San Diego, CA, USA), and the data were analyzed using QIIME version 1.9.0^45^. Low-quality reads with incorrect primer sequences or ambiguous bases were excluded. Using the unique nucleotide barcodes, the reads were classified into groups. The taxonomic assignment was performed based on a 97% similarity with the GreenGenes database (version 13.5) using QIIME. The DNA sequences were deposited in the National Center for Biotechnology Information Short Read Archive under the Accession No. PRJNA655630.

Microbiome compositions were further evaluated using principal component analysis (PCA) plots and compared using the analysis of similarities (ANOSIM) test. Differential abundant features of relative abundances of bacterial taxa were identified using the linear discriminant analysis^46^.

### Sample size calculation

The proportion of complete response was assumed to be 60% because the treatment response in NERD is usually lower than that in erosive esophagitis^1,22^. To detect a difference of 0.2 using a two-sided binomial test, a sample size of 49 is required at a 5% target significance level and 80% statistical power. Considering a 20% dropout rate, we planned to recruit 62 patients in this study.

### Statistical analysis

Continuous and categorical variables were presented as mean with standard deviation and frequency with percentage, respectively. The expression of biologic markers between any two time points was compared by the Wilcoxon singed-rank test. Because paired data were unavailable due to missing values in some patients, OTUs and microbial diversity index between any two time points were compared using the Mann–Whitney U test. The expression of biologic markers and microbial diversity index between patient subgroups were also compared by the Mann–Whitney U test. The correlations between the expression of biologic markers and the relative abundance of bacterial taxa were evaluated using the Pearson correlation test. All *P*-values were two-tailed, and *P*-values of < 0.05 were considered statistically significant. All statistical procedures were conducted using R (version 3.6.2; R Foundation for Statistical Computing, Vienna, Austria), except for linear discriminant analysis, which was performed using Galaxy, an open, web-based platform for computational biomedical research (https://huttenhower.sph.harvard.edu/galaxy).

## Supplementary information

Supplementary Information.
