# Supplementary material for: Treatment of non-erosive reflux disease and dynamics of the esophageal microbiome: a prospective multicenter study
Source: Sci Rep. 2020 Sep 16;10:15154. doi: 10.1038/s41598-020-72082-8 (PMC7494862; doi:10.1038/s41598-020-72082-8)
Supplement: Supplementary file 1 — Supplementary Information. [file 41598_2020_72082_MOESM1_ESM.pdf]

Treatment of non-erosive reflux disease and dynamics of the esophageal microbiome: A prospective multicenter study

Chan Hyuk Park,[1] Seung In Seo,[2] Joon Sung Kim,[3] Sun Hyung Kang,[4] Beom Jin Kim,[5] Yoon Jin Choi,[6] Hyo Joo Byun,[7] Jung-Ho Yoon,[8] and Sang Kil Lee[9] GERD Research Group of the Korean Society of Neurogastroenterology and Motility

[1]Department of Internal Medicine, Hanyang University Guri Hospital, Hanyang University College of Medicine, Guri, Korea

[2]Department of Internal Medicine, Kangdong Sacred Heart Hospital, Hallym University College of Medicine, Seoul, Korea

[3]Division of Gastroenterology, Department of Internal Medicine, College of Medicine, Incheon St Mary's Hospital, the Catholic University of Korea, Seoul, Korea

[4]Department of Internal Medicine, Chungnam National University School of Medicine

[5]Department of Internal Medicine, Chung-Ang University College of Medicine, Seoul, Korea

[6]Department of Internal Medicine, Korea University Guro Hospital, Department of Internal Medicine, Yonsei Institute of Gastroenterology, Severance Hospital, Yonsei University College of Medicine Seoul, Korea

[7]Department of Internal Medicine, Yonsei Institute of Gastroenterology, Severance Hospital, Yonsei University College of Medicine, Brain Korea 21 PLUS Project for Medical Science, Yonsei University, Seoul, Korea

[8]Department of Internal Medicine, Yonsei Institute of Gastroenterology, Severance Hospital, Yonsei University College of Medicine, Brain Korea 21 PLUS Project for Medical Science, Yonsei University, Seoul, Korea

[9]Department of Internal Medicine, Yonsei Institute of Gastroenterology, Severance Hospital, Yonsei University College of Medicine, Brain Korea 21 PLUS Project for Medical Science, Yonsei University, Seoul, Korea

Correspondence to: Sang Kil Lee, MD, PhD

Department of Internal Medicine, Yonsei Institute of Gastroenterology, Severance Hospital, Yonsei University College of Medicine, 50-1 Yonsei-ro, Seoul, 03722 Republic of Korea

Phone: +82-2-2228-1996, Fax: +82-2-393-6884, E-mail: sklee@yuhs.ac

Supplementary information

Figure S1. Operational taxonomic units and diversity.

The number of OTUs and the Chao1 index tended to be lower in the esophagus than in the oropharynx at baseline as well as at week eight. Overall, significant difference was not observed except in the Chao1 index between oropharynx and esophagus at week eight.

Lines indicate the medians with interquartile ranges.

OTU, operational taxonomic unit; PPI, proton pump inhibitor; NS, not significant.

Figure S2. Principal component analysis of the microbiome according to the time-point (baseline vs. week eight) in the (A) oropharynx and (B) esophagus.

There was no significant difference in microbial compositions between the baseline and week eight in the oropharynx (P=0.334) as well as in the esophagus (P=0.920).

The ANOSIM test was used for comparison among the microbial communities.

ANOSIM, analysis of similarities.

Figure S3. Linear discriminant analysis of the relative abundance of bacteria between oropharyngeal and esophageal microbiomes.

Significantly more abundant bacterial taxa in each group are presented. At baseline, Enterobacteriaceae and Chitinophagaceae were more abundant, whereas unclassified Clostridiales Family XIII and Methylobacteriaceae were less abundant in the esophageal microbiome compared to those in the oropharyngeal microbiome. At week eight, there was no difference in the relative abundance of bacterial taxa in the esophageal microbiome compared to those in the oropharyngeal microbiome.

LDA, linear discriminant analysis

Table S1. Treatment responses in patients assessed for biologic markers and microbiomes.

Table S2. Primer sequences for the quantitative polymerase chain reaction for biologic marker analyses.

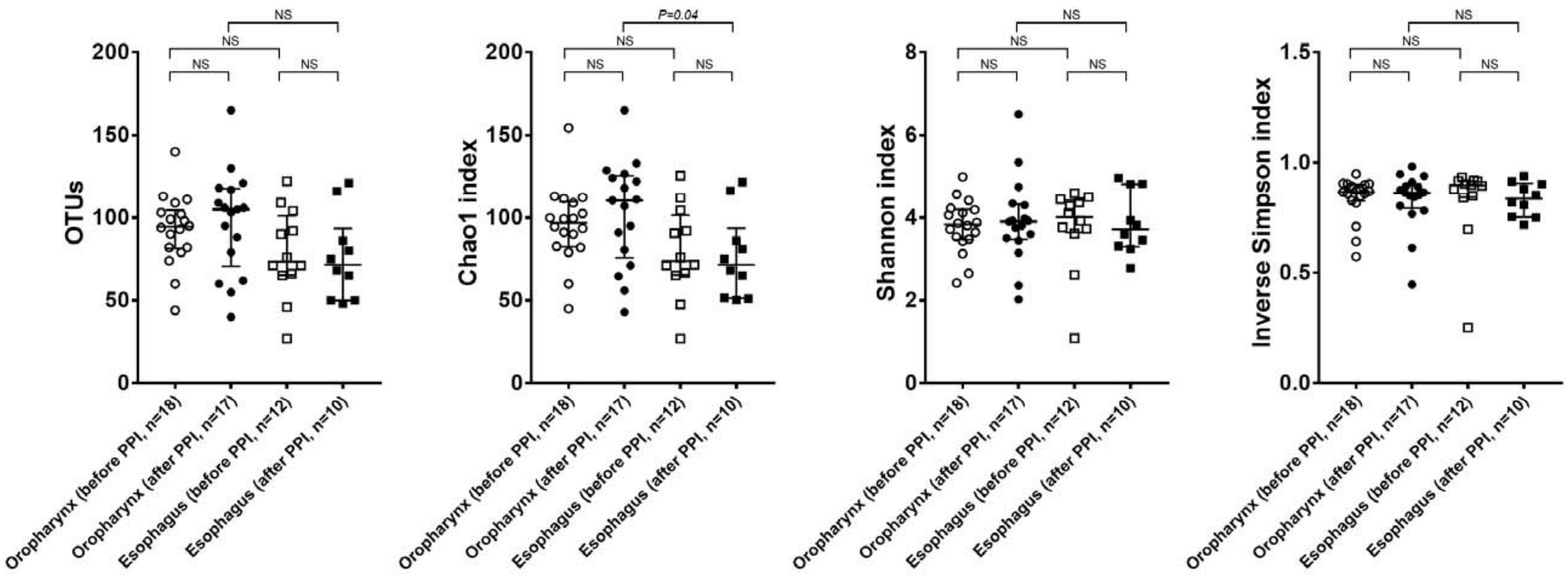

Figure S1. Operational taxonomic units and diversity.

The number of OTUs and the Chao1 index tended to be lower in the esophagus than in the oropharynx at baseline as well as at week eight. Overall, significant difference was not observed except in the Chao1 index between oropharynx and esophagus at week eight.

Lines indicate the medians with interquartile ranges.

OTU, operational taxonomic unit; PPI, proton pump inhibitor; NS, not significant.

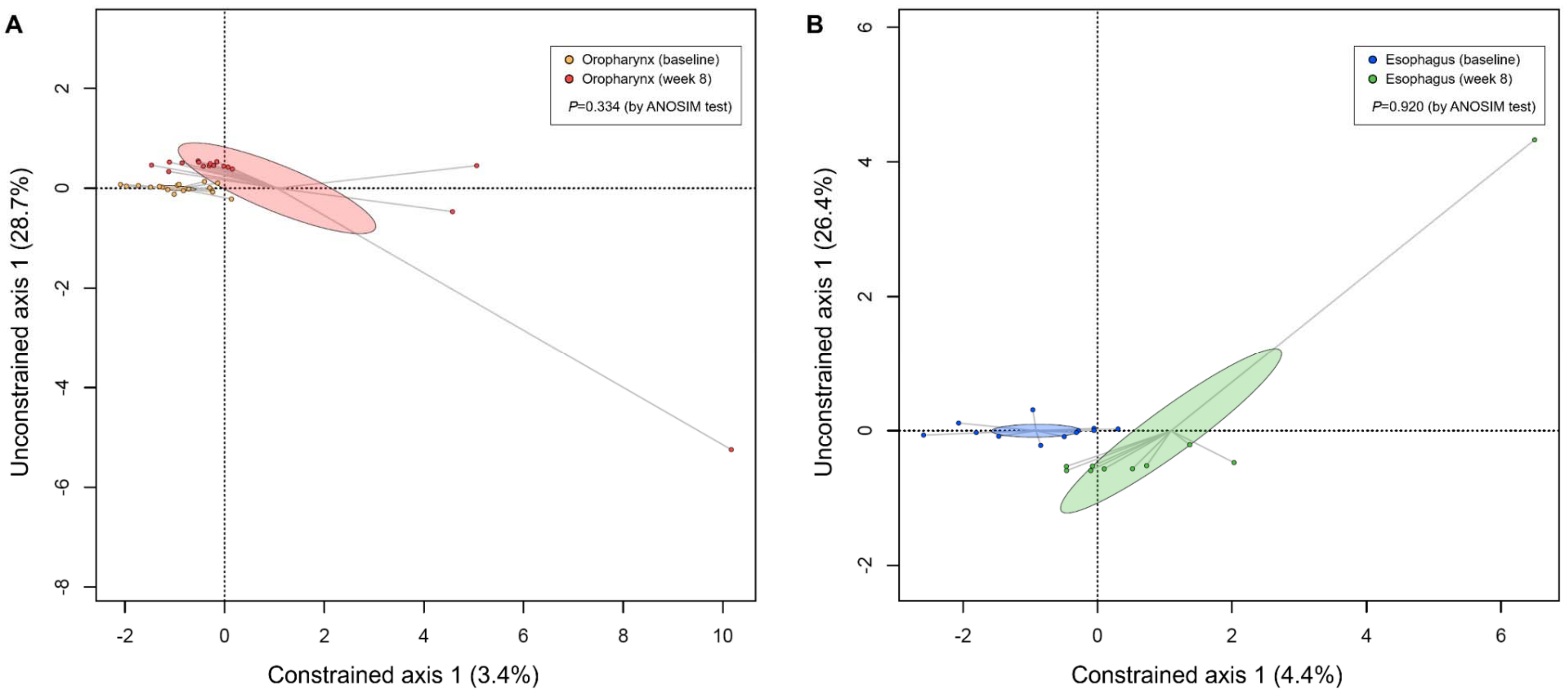

Figure S2. Principal component analysis of the microbiome according to the time-point (baseline vs. week eight) in the (A) oropharynx and (B) esophagus.

There was no significant difference in microbial compositions between the baseline and week eight in the oropharynx (*P*=0.334) as well as in the esophagus (*P*=0.920).

The ANOSIM test was used for comparison among the microbial communities.

ANOSIM, analysis of similarities.

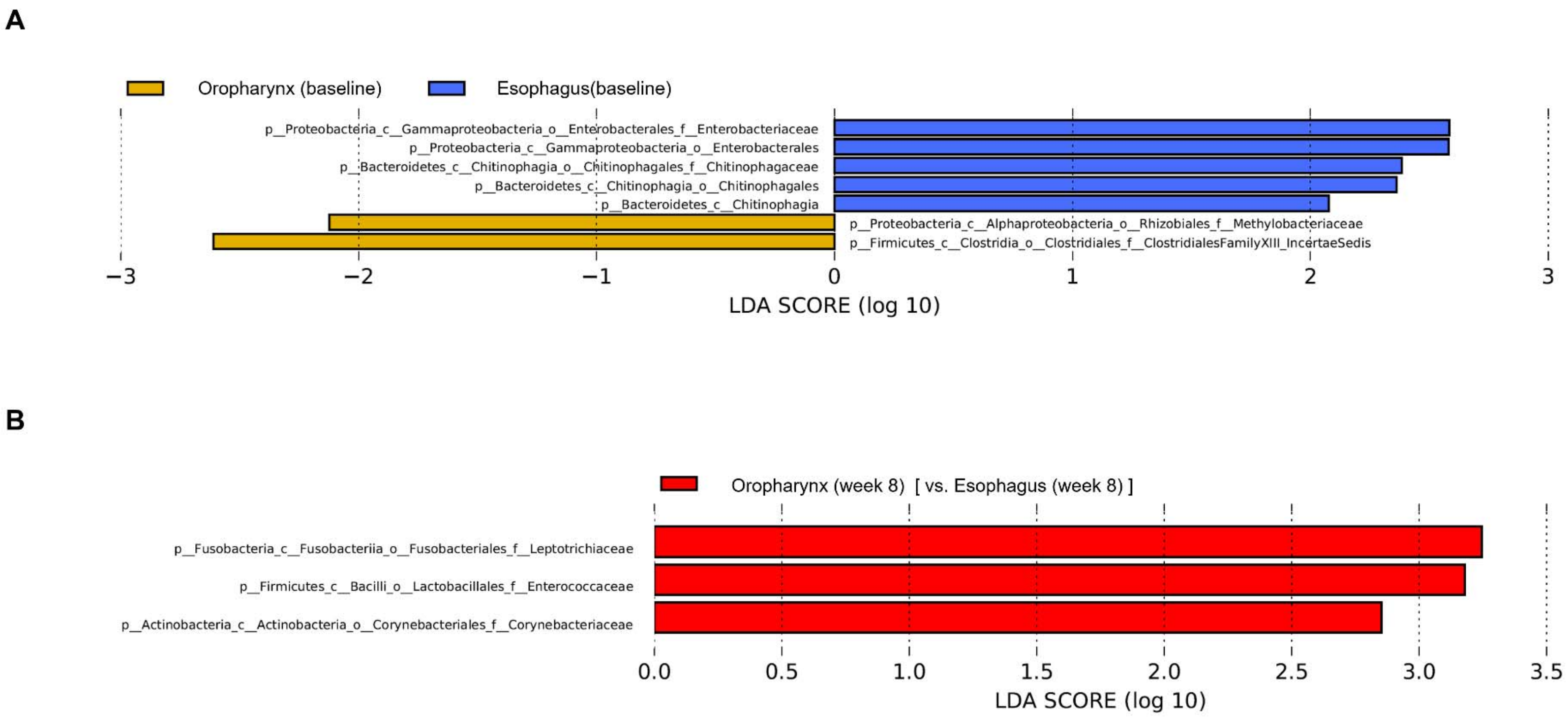

Figure S3. Linear discriminant analysis of the relative abundance of bacteria between oropharyngeal and esophageal microbiomes.

Significantly more abundant bacterial taxa in each group are presented. At baseline, Enterobacteriaceae and Chitinophagaceae were more abundant, whereas unclassified Clostridiales Family XIII and Methylobacteriaceae were less abundant in the esophageal microbiome compared to those in the oropharyngeal microbiome. At week eight, there was no difference in the relative abundance of bacterial taxa in the esophageal microbiome compared to those in the oropharyngeal microbiome.

LDA, linear discriminant analysis

Table S1. Treatment responses in patients assessed for biologic markers and microbiomes.

Variable	Value
N	18
Treatment response to PPI at week 8	
Heartburn	
Complete response	10 (55.6)
Partial response	7 (38.9)
Non-response	1 (5.6)
Regurgitation	
Complete response	9 (50.0)
Partial response	6 (33.3)
Non-response	3 (16.7)

BMI, body mass index; PPI, proton pump inhibitors; SD, standard deviation

Table S2. Primer sequences for the quantitative polymerase chain reaction for biologic marker analyses.

Type of biologic marker	Direction	Sequences (5' to 3')
Inflammatory marker		
IFN-γ	Forward	GGCTTAATTCTCTCGGAAACG
	Reverse	ATGGGTCCTGGCAGTAACAG
IL-1β	Forward	CCACAGACCTTCCAGGAGAATG
	Reverse	GTGCAGTTCAGTGATCGTACAGG
IL-6	Forward	GTTGTGCAAGGGTCTGGTTT
	Reverse	TTCCCTCAGGATGGTGTCTC
IL-8	Forward	CAAGAGCCAGGAAGAAACCA
	Reverse	AGCTGCAGAAATCAGGAAGG
IL-10	Forward	TGCAAAACCAAACCACAAGA
	Reverse	TGCAGCTGTTCTCAGACTGG
MCP1	Forward	CCCAGAAACATCCAATTCTCA
	Reverse	GGAATGAAGGTGGCTGCTAT
NF-κB	Forward	TGGAGTCTGGGAAGGATTTG
	Reverse	CGAAGCTGGACAAACACAGA
RANTES	Forward	CGCTGTCATCCTCATTGCTA
	Reverse	GCACTTGCCACTGGTGTAGA
TNF-α	Forward	AAAAATGGGCTCCCTCTCATCAGTTC
	Reverse	TCTGCTTGGTGGTTTGCTACGAC
Permeability marker		
OCLN	Forward	TTTGTGGGACAAGGAACACA
	Reverse	TAGTCAGATGGGGGTGAAGG
ZO-1	Forward	CCGTTGGCATGAAGTGTATG
	Reverse	AGCCAGACCTGCAAGAAGAA
CLDN-1	Forward	GCCCCAGTGGAGGATTTACT
	Reverse	TGCTCAGATTCAGCAAGGAG
CLDN-4	Forward	CTGTGGCCTCAGGACTCTCT
	Reverse	ACCCTCCCAGGCTCATTAGT
Hypersensitivity marker		
ASIC-3	Forward	GCCGTCACCAAGACTCTCTC
	Reverse	TAGGCATGTCCAGGATGTCA
TRPV1	Forward	GCCTGGAGCTGTTCAAGTTC
	Reverse	GATGAGCATGTTGAGCAGGA
Control		
GAPDH	Forward	CCGGGAAACTGTGGCGTGATGG
	Reverse	AGGTGGAGGAGTGGGTGTCGCTGTT
